# Serum short-chain fatty acids and its correlation with motor and non-motor symptoms in Parkinson’s disease patients

**DOI:** 10.1186/s12883-021-02544-7

**Published:** 2022-01-07

**Authors:** Gang Wu, Zhengli Jiang, Yaling Pu, Shiyong Chen, Tingling Wang, Yajing Wang, Xiaoping Xu, Shanshan Wang, Minya Jin, Yangyang Yao, Yang Liu, Shaofa Ke, Suzhi Liu

**Affiliations:** 1 Department of Pharmacy, Taizhou Hospital of Zhejiang Province affiliated to Wenzhou Medical University, Linhai, 317000 Zhejiang China; 2 Department of Neurology, Taizhou Hospital of Zhejiang Province affiliated to Wenzhou Medical University, Linhai, 317000 Zhejiang China; 3 Clinical Medical College, Shaoxing University of Arts and Sciences, Shaoxing, 312099 Zhejiang China; 4 Clinical laboratory Department, Taizhou Hospital of Zhejiang Province affiliated to Wenzhou Medical University, Linhai, 317000 Zhejiang China; 5 Health Management Center, Taizhou Hospital of Zhejiang Province affiliated to Wenzhou Medical University, Linhai, 317000 Zhejiang China; 6 Department of Neurology, Saarland University, 66421 Homburg, Germany

**Keywords:** Short-Chain Fatty Acids, Parkinson’s Disease, Cognitive Impairment, Depression, Propionic Acid, trihexyphenidyl, tizanidine

## Abstract

**Background:**

Parkinson’s disease (PD) is associated with enteric nervous system dysfunction and gut microbiota dysbiosis. Short-chain fatty acids (SCFAs), derived from gut microbiota, are supposed to anticipate PD pathogenesis via the pathway of spinal cord and vagal nerve or the circulatory system. However, the serum concentration of SCFAs in PD patients is poorly known. This study aims to investigate the exact level of SCFAs in PD patients and its correlation with Parkinson’s symptoms.

**Methods:**

50 PD patients and 50 healthy controls were recruited, and their demographic and clinical characteristics were collected. The serum concentration of SCFAs was detected using a gas chromatography-mass spectrometer. SCFAs were compared between PD and control groups. The correlation between serum SCFAs and Parkinson’s symptoms and the potential effects of medications on the serum SCFAs was analyzed.

**Results:**

Serum propionic acid, butyric acid and caproic acid were lower, while heptanoic acid was higher in PD patients than in control subjects. However, only the serum level of propionic acid was correlated with Unified Parkinson’s Disease Rating Scale (UPDRs) part III score (R = -0.365, *P* = 0.009), Mini-mental State Examination (MMSE) score (R = -0.416, *P* = 0.003), and Hamilton Depression Scale (HAMD) score (R = 0.306, *P* = 0.03). There was no correlation between other serum SCFAs and motor complications. The use of trihexyphenidyl or tizanidine increased the serum concentration of propionic acid.

**Conclusions:**

Serum SCFAs are altered in PD patients, and the decrease of serum propionic acid level is correlated with motor symptoms, cognitive ability and non-depressed state. Thus, the gut microbial-derived SCFAs potentially affect Parkinson’s symptoms through the blood circulation. Propionic acid supplementation might ameliorate motor and non-motor symptoms of PD patients, although clinical trials are needed to test this hypothesis.

**Supplementary Information:**

The online version contains supplementary material available at 10.1186/s12883-021-02544-7.

## Background

Short-chain fatty acids (SCFAs) are saturated aliphatic organic acids with one to six carbons, and produced by gut microbiota through fermentation of dietary fiber [1]. After being produced and absorbed in the gut, SCFAs are transported to the liver, and some of them enter the systemic blood circulation system [2]. The gut microbiota is disturbed in patients of Parkinson’s disease (PD), which is associated with motor or non-motor symptoms [3–8]. SCFAs or metabolites from gut microbiota are also changed in the feces or serum of PD patients [9, 10]. SCFAs have extensive physical effects on cellular energy metabolism, cholesterol biosynthesis, anti-inflammatory, and immune system regulation [11]. However, it remains debated whether SCFAs modulate the function of central nervous system through interacting with gastrointestinal, vagal nerves and spinal cord or directly acting on brain cells [12, 13]. Recently, activation of free fatty acid receptor 3 (FFAR3) was reported to attenuate the motor deficits and dopaminergic neuronal loss in a 6-hydroxydopamine-induced PD mouse model [14]. In this study, we aimed to determine the concentration of SCFAs in the serum of PD patients and investigate the relationship between serum SCFAs and Parkinson’s symptoms.

## Methods

### Subjects

PD patients were recruited from Taizhou Hospital of Zhejiang Province from July, 2020 to January, 2021. The inclusion criteria were: (1) agreement to participate in the research; (2) aged between 60 and 75 years; (3) diagnosis of PD according to Diagnostic criteria of Parkinson’s disease in China (2016 edition) [15]; (4) no use of antibiotics for three months; (5) no use of omega-3, probiotics for two weeks; (6) no use of lipid-lowering medicine for one month. The healthy controls were recruited from Health Management Center at Taizhou Hospital of Zhejiang Province from July, 2020 to January, 2021 and met the above inclusion criteria except diagnosis of PD.

Exclusion criteria were: (1) secondary parkinsonism, atypical parkinsonism, Alzheimer’s disease, cerebrovascular disease or other central nervous system diseases; (2) celiac disease; (3) chronic pancreatitis; (4) history of gastrointestinal surgery; (5) inflammatory bowel disease; and (6) history of cancer within three years.

### Clinical evaluation

All participants provided demographics of age, sex, smoke, alcohol consumption, hypertension, diabetes mellitus, liver function index, lipid profiles, and medical history. For all patients, the following data were recorded: disease duration from onset to study, Hoehn-Yahr stage, motor symptom related-Unified Parkinson’s Disease Rating Scale (UPDRS) part III score, motor complications (end-of-dose phenomenon, dyskinesia, and freezing), non-motor symptoms (cognitive impairment, anxiety, depression, paresthesia, dysautonomia, sleep disorders and rapid-eye-movement sleep behavior disorder (RBD)), medication usage and Levodopa-equivalent daily dose (LEDD). The UPDRS scores were rated during the on state. Motor complications and paresthesia were evaluated by clinical face-to-face interviews with patients. Sleep disorders was evaluated according to Parkinson disease sleep scale. Autonomic function was evaluated using the Scale for Outcomes in Parkinson’s Disease-Autonomic Dysfunction. Patients that often had symptoms related to postural changes, urinary dysfunction caused by non-primary or secondary causes of the urinary system, or constipation, were considered to have dysautonomia. The cognitive state was evaluated using Mini-mental State Examination (MMSE) scale. The anxiety and depression states were evaluated using Hamilton Anxiety Scale (HAMA) and Hamilton Depression Scale (HAMD), respectively. RBD was diagnosed with REM Sleep Behavior Disorder Questionnaire Hong Kong (RBDQ-HK). All the assessments were performed by two experienced physicians specialized in movement disorders (Suzhi Liu and Yajing Wang, with 21 and 10 years of work experience on Parkinson’s Disease, respectively). LEDD was calculated according to the reference [16].

### Collection of serum samples

Blood was sampled into serum-separating tubes by professional nurses in the morning after overnight fasting. After 30 min at the room temperature, the blood samples were centrifuged at 4000 rpm for 5 min at 4 °C. The serum was collected into a 1.5 mL Eppendorf tube and stored at −80 °C until assay.

### Determination of SCFAs concentrations

Concentrations of serum SCFAs (including heptanoic acid) were analyzed using a gas chromatography-mass spectrometer (GC-MS). Serum samples were deproteinized by phosphoric acid and extracted with ether. After being centrifuged at 4000 rpm for 10 min, the supernatant was collected and injected to GC-MS. An Agilent 7890B-7000D GC-MS was fitted with a capillary column HP-INNOWAX 25 m × 0.20 mm × 0.4 μm. The injector temperature was 240 °C, and the carrier gas flow rate was set to 1 mL/min. The ion source and transmission line temperatures were 200 and 250 °C, respectively. The electron bombarding voltage was 70 eV, and single ion monitoring was applied.

### Statistical analysis

Continuous variables were expressed as mean ± Std.Error and compared using student’s t-test if data were normally distributed or otherwise, expressed as median (IQR) and analyzed using Mann-Whitney U test. The Kolmogorov-Smirnov test was used to evaluate the normality of the distribution of the variables. Categorical variables were expressed as number (%) and compared by χ2 test or Fisher’s exact test. A correlation was investigated using Spearman nonparametric correlation analysis method. Statistical significance values were set at α ≤ 0.05 (two-sided), and correlation magnitude levels were defined as weak (<0.3), moderate (0.3–0.59), and strong (≥0.6). Data analysis was conducted using SPSS software, version 16.0 (SPSS Inc.). Multiple testing of the nine SCFAs between PD and controls was then corrected by Bonferroni method. The potential confounding variables predictive of SCFAs was determined by multiple linear regression using stepwise method.

## Results

### Clinical and laboratory characteristics of participants

50 PD patients and 50 normal controls were recruited in this study. The main clinical characteristics and laboratory results are described in Table 1. No difference was observed in age and gender between PD patients and controls (*P* = 0.450 and 0.675 respectively). No difference existed in the smoke and alcohol consumption rate between PD patients and controls (*P* = 0.269 and 0.359 respectively). PD and controls exhibited the same rate of hypertension and diabetes mellitus comorbidity (both *P* = 1.000). The two groups exhibited no difference in laboratory characteristics, including bilirubin, creatinine, total cholesterol, triglycerides, or high-density lipoprotein cholesterol. However, low-density lipoprotein cholesterol in PD group was significantly lower than in the control group (mean ± Std.Error, PD vs. control, 2.91 ± 0.09 vs 2.47 ± 0.12, *P* = 0.005) (Table 1).

**Table 1:** Clinical and laboratory characteristics of the participants

	Control(n = 50)	PD(n = 50)	*P* value
Age, median (IQR), y	66.5(6)	68.0(5)	0.450
Male,n(%)	34(68.0)	31(62)	0.675
Smoke,n(%)	6(12.0)	2(4.0)	0.269
Alcohol consumption,n(%)	4(8.0)	1(2.0)	0.359
Hypertension,n(%)	12(24.0)	12(24.0)	1.000
Diabetes mellitus,n(%)	7(14.0)	8(16.0)	1.000
Bilirubin,median (IQR),median (IQR),μmol/L	16.20(6.90)	15.70(9.12)	0.728
Creatinine,mean ± Std.Error,μmoI/L	70.78 ± 1.66	69.08 ± 2.12	0.530
Total cholesterol,mean ± Std.Error,mmol/L	4.99 ± 0.12	4.64 ± 0.14	0.065
Triglycerides,median (IQR),mmol/L	1.31(0.96)	1.23(1.19)	0.463
High-density lipoprotein cholesterol,mean ± Std.Error,mmol/L	1.53 ± 0.05	1.49 ± 0.04	0.558
Low-density lipoprotein cholesterol,mean ± Std.Error,mmol/L	2.91 ± 0.09	2.47 ± 0.12	0.005
Disease duration,median (IQR),m		51.5(48)	
Hoehn-Yahr stage		2.5(0.5)	
UPDRS part III score,mean ± Std.Error		42.82 ± 2.20	
MMSE score,mean ± Std.Error		20.24 ± 0.73	
Motor complications, n(%)			
End-of-dose phenomenon		25(50.0)	
Dyskinesia		7(14.0)	
Freezing		16(32.0)	
Non-motor symptoms			
HAMA score,median (IQR)		15(14.0)	
HAMD score,median (IQR)		11.5(12.0)	
Paresthesia,n(%)		46(92.0)	
Dysautonomia,n(%)		48(98.0)	
Sleep disorders,n(%)		36(72.0)	
RBD,n(%)		20(40.0)	
Drug therapy,n(%)			
LEDD(mg)		555.5 ± 38.9	
Levodopa(levodopa and serazide or kazodidopa)		38(76.0)	
Dopamine agonist(pramipexole,pyrenoid or bromocriptine)		31(62.0)	
MAOI(selegiline or rasagiline)		9(18.0)	
Amantadine		25(50.0)	
Trihexyphenidyl		14(28.0)	
Tizanidine		16(32.0)	

*P* values comparing control and PD participants are from Mann-Whitney U test, χ^2^ test, or Student’s t test. *IQR* interquartile range, *UPDRS* Unified Parkinson’s Disease Rating Scale, *MMSE* Mini-mental State Examination, *HAMA* Hamilton Anxiety Scale, *HAMD* Hamilton Depression Scale, *RBD* REM sleep behavior disorder, *MAOI* monoamine oxidase inhibitor

PD patients had a disease duration of 51.5(48) (median (IQR)) months and the Hoehn-Yahr stage was 2.5(0.5). The UPDRS part III score was 42.82 ± 2.20 (mean ± Std.Error) and MMSE score was 20.24 ± 0.73 (mean ± Std.Error). In addition, patients with longer disease duration appeared to have higher UPDRS part III score (R = 0.494, *P* = 0.000) (Supplementary Fig. 1). The prevalence of motor complications such as end-of-dose phenomenon, dyskinesia, and freezing was described. The prevalence of non-motor symptoms, including anxiety, depression, paresthesia, dysautonomia, sleep disorders and RBD in PD patients was calculated. The usage of anti-Parkinson’s agent was also calculated and LEDD is 555.5 ± 38.9 mg (Table 1).

### The levels of serum SCFAs in PD patients and healthy controls

**Fig. 1:**
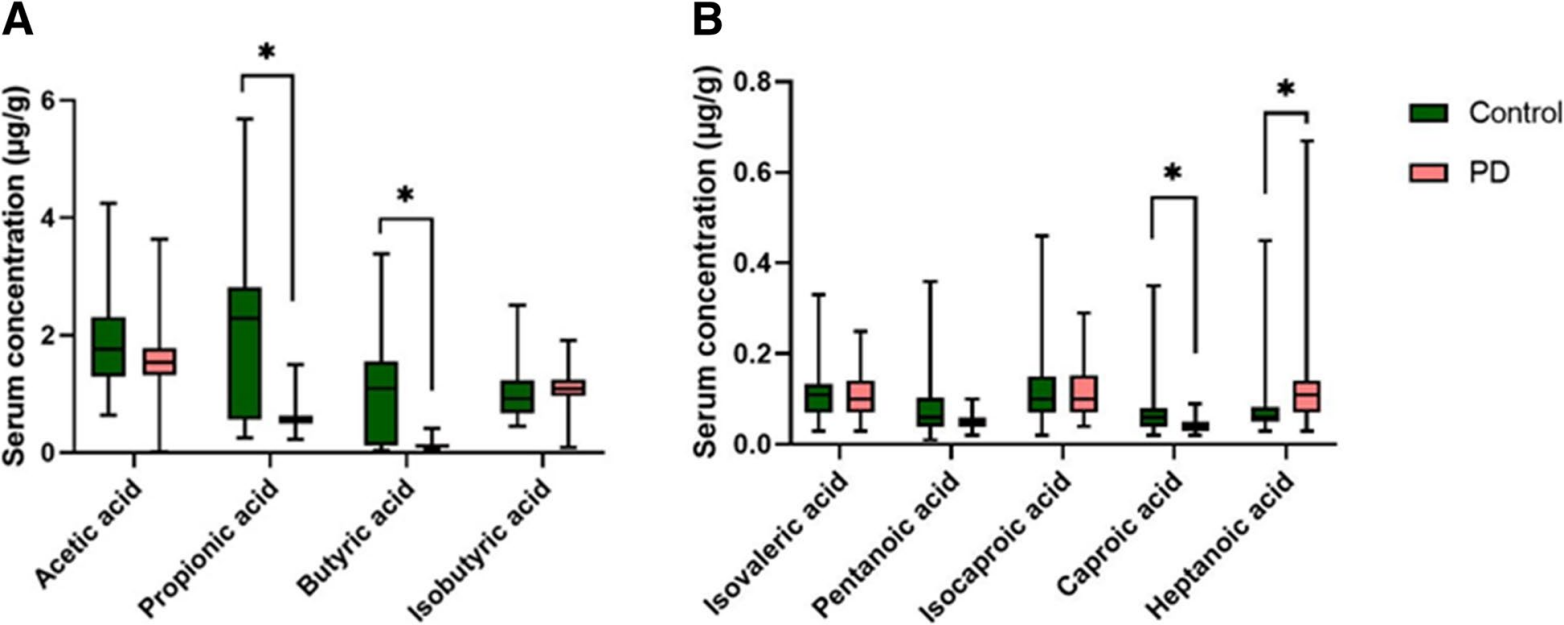
Comparison of peripheral serum concentrations of acetic acid, propionic acid, butyric acid, and isobutyric acid (**A**) as well as isovaleric acid, pentanoic acid, isocaproic acid, caproic acid, and heptanoic acid (**B**) between PD and control participants. Green represents control (n = 50) and red is PD patients (n = 50). *, *P* < 0.0056 (0.05/9)

The levels of serum propionic, butyric and caproic acids were significantly lower in PD group than in control group after correction by Bonferroni method (*P* < 0.0056 (0.05/9)). Heptanoic acids were significantly higher in PD patients than controls (*P* < 0.0056). No differences were observed between the two groups in pentanoic acid (*P* = 0.008), isobutyric acid (*P* = 0.019), acetic acid (*P* = 0.072), isovaleric acid (*P* = 0.450), or isocaproic acid (*P* = 0.937) (Fig. 1).

Although demographic variables of PD and control group were similar, multiple linear regression using stepwise method was applied to determine the explanatory variables predictive of SCFAs. The model showed that: 1) PD (*P* < 0.000), smoke (*P* = 0.022), triglycerides (*P* = 0.019) and creatinine (*P* = 0.047) were significant predictors for the decrease of propionic acid, accounting for 39.6% (*P* = 0.000) of the variance indicated by the adjusted *R*-squared; 2) PD (*P* < 0.000), alcohol consumption (*P* < 0.001) and low-density lipoprotein cholesterol (*P* = 0.044) were significant predictors for the decrease of butyric acid, accounting for 42.3% (*P* = 0.000) of the variance; 3) PD (*P* = 0.005), alcohol consumption (*P* = 0.005) and high-density lipoprotein cholesterol (*P* = 0.038) were significant predictors for the decrease of caproic acid, accounting for 15.2% (*P* = 0.000) of the variance; 4) PD (*P* = 0.021) was also a significant predictor for the increase of heptanoic acid, accounting for 4.3% (*P* = 0.021) of the variance.

### Correlation of serum SCFAs with motor symptoms in PD

**Fig. 2:**
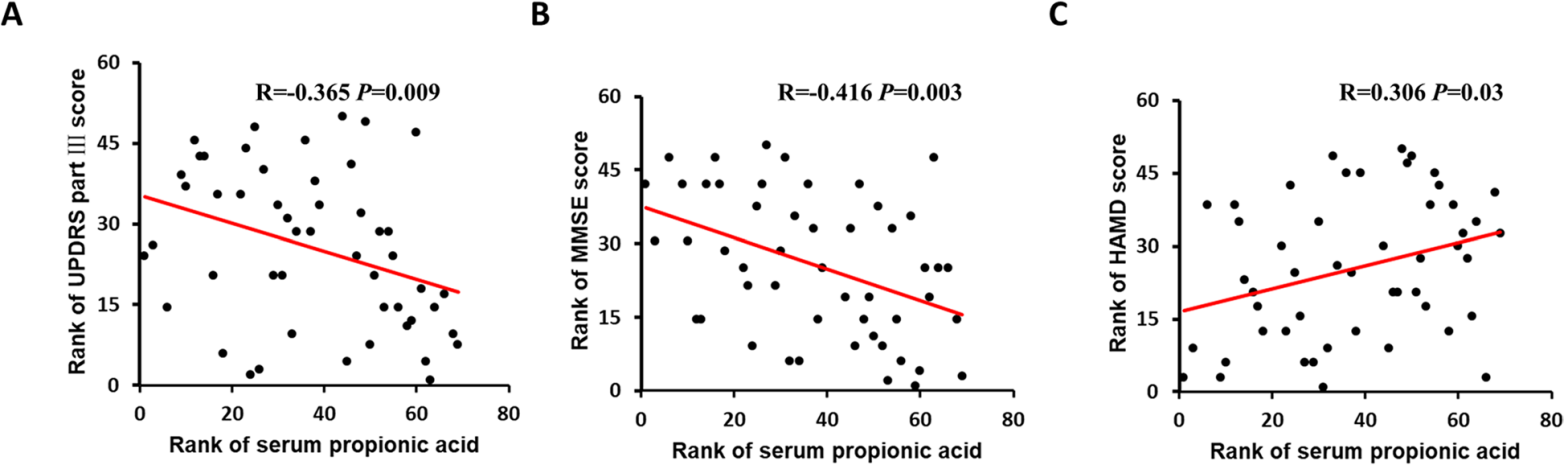
Correlation between serum propionic acid and Parkinson’s symptoms. **A** Serum propionic acid is significantly negatively correlated with UPDRS part III score in PD patients (n = 50), R = -0.365, *P* = 0.009. **B** Scatterplots show a negative correlation between propionic acid concentration and MMSE score, R = -0.416, *P* = 0.003. (C) Scatterplots show a positive correlation between propionic acid concentration and HAMD score, R = 0.306, *P* = 0.03. Spearman nonparametric correlation test was used in the correlation analysis. MMSE, Mini-mental State Examination. HAMD, Hamilton Depression Scale. UPDRS, Unified Parkinson’s Disease Rating Scale

In order to evaluate the relationship between SCFAs and PD progression, the contents of SCFAs in different Hoehn-Yahr stages were analyzed. As there were only 2 and 1 patient in stage 1 and 4 respectively, patients at stage 1 and stage 2, and patients at stage 3 and stage 4 were pooled in the analysis. Between these two pooled groups of patients (stage 1–2 vs. 3–4), the levels of serum SCFAs were not significantly different (p > 0.05). The motor symptom in PD was evaluated by UPDRS part III. We observed that only the serum level of propionic acid was correlated with UPDRS part III scores (Supplementary Table 1). The correlation coefficient of propionic acid with UPDRS part III scores was −0.365, and *P*-value was 0.009, indicating moderate correlation magnitude levels (Fig. 2A).

### Correlation of serum SCFAs with non-motor symptoms and motor complications in PD

After we observed a correlation between serum SCFA and motor symptoms in PD patients, we analyzed the potential relation between SCFAs and non-motor symptoms. We observed that only the level of serum propionic acid was correlated with cognitive impairment and depression. As shown in Fig. 2, B and C, serum propionic acid was negatively correlated with MMSE score (R = -0.416, *P* = 0.003), but positively correlated with HAMD score (R = 0.306, *P* = 0.03). Notably, HAMD score was negatively correlated with MMSE score (R = -0.375, *P* = 0.007) (Supplementary Fig. 2), implying that depressed patients exhibit cognitive impairment or vice versa.

No correlation was observed between SCFAs and motor complications. The serum SCFAs concentration was comparable in patients with and without motor complications (data not shown).

### Relation of anti-Parkinson medication to serum SCFAs

**Fig. 3:**
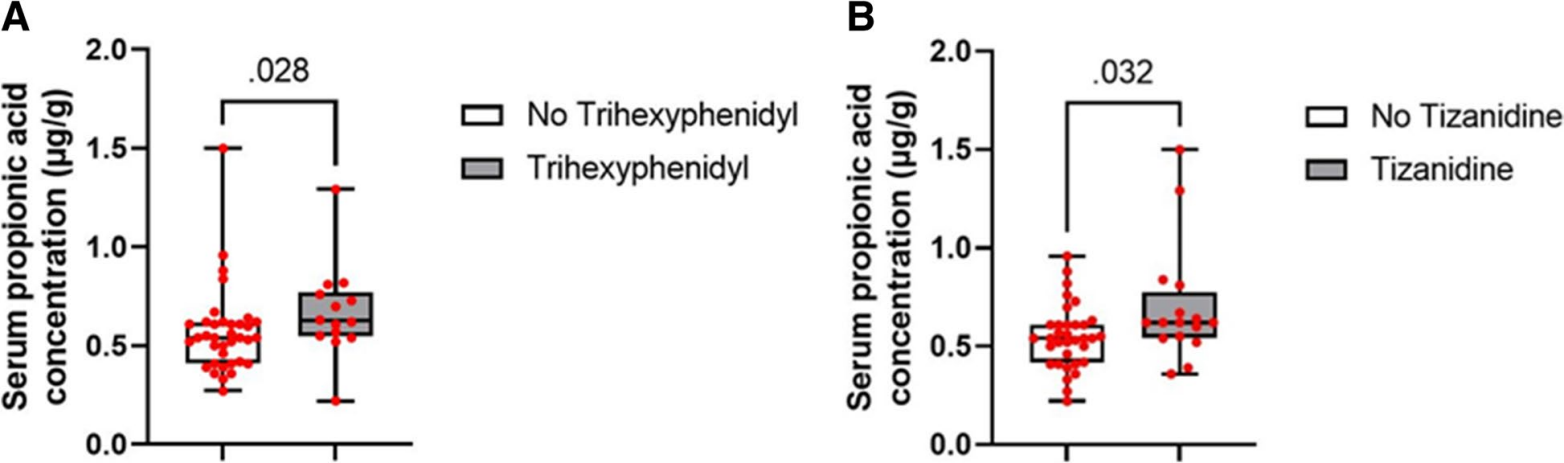
The effect of anti-Parkinson medications on peripheral venous concentrations of SCFAs in PD patients. **A** Serum propionic acid concentration was higher in PD patients taking trihexyphenidyl (n = 14) (*P* = 0.028). **B** Serum propionic acid concentration was higher in PD patients taking tizanidine (n = 16) (*P* = 0.032)

All PD patients were administered with anti-PD drug. To investigate the influence of anti-Parkinson medication on peripheral blood SCFAs, the serum SCFAs concentration was compared between people who received a specific anti-Parkinson medication and those who did not receive it. The results showed that the serum SCFAs appeared not to be influenced by levodopa administration as correlation between SCFAs and LEDD was not observed. However, serum propionic acid concentration was significantly higher in PD patients taking trihexyphenidyl (n = 14) (*P* = 0.028) (Fig. 3A) or tizanidine (n = 16) (*P* = 0.032) (Fig. 3B). Other anti-Parkinson medication’s influence on serum SCFAs concentration was not observed.

## Discussion

Gastrointestinal symptoms often appear prior to motor symptoms. Current researches have demonstrated intestinal microbial disturbances in PD patients [3, 17]. The disorder of intestinal flora is not only related to Parkinson’s motor phenotype [7], but also to its non-motor symptoms [5, 6, 8]. It is hypothesized that intestinal flora and their metabolites play a direct role in the pathogenesis of PD [17, 18]. SCFAs are derived from gut microbial metabolism and alter brain function not only through gastrointestinal, spinal cord, and vagal nerves but also by directly interacting with brain cells after circulating in the blood system [12, 13, 19, 20]. However, the exact concentration of SCFAs in the blood of PD patients and its association with Parkinson’s symptoms are unclear. To the best of our knowledge, this study is the first research to examine SCFAs in serum specimens from PD patients. Moreover, we investigated the correlation of serum SCFAs with motor or non-motor symptoms, as well as motor complications of PD.

The results indicated that clinical and laboratory characteristics remained relatively consistent between PD patients and healthy controls. We observed that levels of propionic acid, butyric acid and caproic acid decreased, while, the level of heptanoic acid increased in PD patients compared with healthy controls. The differences in propionic acid and butyric acid are in line with the alterations of gut microbes in PD. In the feces of PD patients, the abundance of *Prevotella*, which produces propionate, decreases [21], and the abundance of putative-butyrate–producing bacteria, such as *Faecalibacterium*, *Prausnitzii*, *Blautia*, *Coprococcus*, *Roseburia*, and *Eubacterium*, also decreases [1, 4, 9–11]. Thus, the decreased propionic acid and butyric acid in the serum might arise from alteration of intestinal flora. Numerous studies have identified increased abundance of putative-acetate-producing bacteria, such as *Bifidobacterium*, *Lactobacillus*, *Clostridium clusters*, and *Akkermansia muciniphila*, reduced abundance of *Prevotella*, *Bacteroides*, *Blautia*, *Clostridium* spp., and *Ruminococcus* [1, 11] in Parkinson’s disease. The overall equilibrium of putative-acetate-producing bacteria might result in the same amount of metabolized acetic acid and subsequent serum acetic acid in Parkinson patients as controls in present study.

Nutrient intake was supposed to regulate the generation of SCFAs and modify PD pathogenesis [22, 23]. It was reported that PD patients had a high intake of dietary fiber, which is the source of SCFAs [24]. In our study, we observed that most types of SCFAs decrease in PD patients. Thus, the abnormal serum SCFAs is mainly due to the intestinal microbial disorders instead of nutrient patterns of PD patients.

We observed that propionic acid in the serum was moderately negatively correlated with UPDRS part III score of PD patients, which is consistent with a previous study [25]. Although propionic acid might act on FFAR3 in the gut and ameliorate motor deficits and dopaminergic neuron loss in 6-hydroxydopamine-induced PD mice [14], it is possible that the circulating propionic acid directly protects neurons in the brain. An *in vitro* experiment showed that treatment with propionic acid prevented dopaminergic neurons from the neurotoxicity of rotenone and enhanced the outgrowth of neurites [26]. Moreover, as an HDAC inhibitor [11], propionic acid might also inhibit the neuroinflammatory activation and attenuate the damage of blood-brain-barrier [27], the two characteristic pathological changes in PD brain [28].

Moreover, propionic acid in the serum was negatively correlated with MMSE score, suggesting that lower serum propionic acid is linked to normal cognitive ability in PD. It was reported that the abundance of genus *Ruminococcus*, a putative propionic acid producer, was decreased significantly in the moderate cognitive impairment group than that of a normal cognitive group in PD patients. In addition, its abundance negatively correlated with cognition ability in PD [8]. It has been shown that patients with propionic acidemia often display cognitive deficits [29]. A chronic subcutaneous injection of propionic acid induces cognitive dysfunction in adult rats [30]. Moreover, the serum level of propionic acid was positively correlated with HAMD score, which suggests that propionic acid affects the depression status of PD patients. Family Ruminococcaceae and fecal propionic acid decrease in depressed mice compared to control mice [31], however, intraperitoneal injection of propionic acid at a low dose could inhibit the social motivation of rats [32]. Thus, the administration route should be considered and evaluated if propionic acid is supplemented to PD patients [33].

The microbial metabolism affects the pharmacokinetics of neuromodulatory drugs, and on the other way around, medication can alter gut microbiota composition and SCFAs production [34, 35]. Indeed, administration of anti-PD drugs, trihexyphenidyl and tizanidine, increased the serum concentration of propionic acid. However, since the gut microbiota was not identified in this study, a clear relationship between anti-PD therapy, gut microbiota, and SCFAs could not be clarified. As described above, serum propionic acid is related to cognitive impairment and depression in PD, however, treatments of trihexyphenidyl or tizanidine were not correlated with MMSE or HAMD scores (data not shown).

It is similar with previous studies [36–39] that the levels low-density lipoprotein cholesterol decreases in PD patients. We observed that low-density lipoprotein cholesterol was weakly correlated with acetic, propionic, and butyric acids in the serum of all participants including PD patients and healthy controls; however, in the separate PD group, the serum level of low-density lipoprotein cholesterol was not correlated with any SCFAs, UPDRS part III score, non-motor symptoms or motor complications (data not shown). Thus, low-density lipoprotein cholesterol dose not interfere with the effects of serum SCFAs levels on Parkinson’s symptoms, although it mignt interact with SCFAs.

There are three limitations in this study: 1, the sample size was small, which limited the number of patients with Hoehn-Yahr stages 1 and 4; and 2, the fecal microbiota and SCFAs were not analyzed, which made it unclear how the gut bacteria affect serum SCFAs; and 3, serum SCFAs in drug naive PD patients and their correlation with Parkinson’s symptoms were not investigated. A more comprehensive study is still needed.

## Conclusions

In summary, our study provides additional evidence for the alteration of serum SCFAs in PD patients. We further observed that the serum level of propionic acid was decreased and associated with both motor and non-motor symptoms (cognitive dysfunction and depression status of PD patients). In addition, we found that some anti-Parkinson medications such as trihexyphenidyl and tizanidine affected serum propionic acid. Our study supports that the gut bacteria-derived SCFAs could act on brain cells through circulating in the blood stream. Clinical trials are needed to investigate whether the supplement of propionic acid could improve the motor symptoms and mental functions of PD patients.

## Supplementary Information

**Additional file 1: Supp Table 1.** The correlation between serum SCFAs and UPDRS part III score. **Supplementary Fig. 1.** UPDRS part III score was significantly positively correlated with disease duration in PD patients (n = 50), R = 0.494, *P* = 0.000. Investigated by Spearman nonparametric correlation analysis method. UPDRS, Unified Parkinson’s Disease Rating Scale. **Supplementary Fig. 2.** MMSE score was significantly negatively correlated with HAMD score in PD patients, R = -0.375, *P* = 0.007. Investigated by Spearman nonparametric correlation analysis method. MMSE, Mini-mental State Examination. HAMD, Hamilton Depression Scale.

## Data Availability

The datasets used and/or analysed during the current study are available from the corresponding author on reasonable request.

## Abbreviations

FFAR: Free Fatty Acid Receptor
GC-MS: Gas Chromatography-Mass Spectrometer
HAMA: Hamilton Anxiety Scale
HAMD: Hamilton Depression Scale
HDAC: Histone Deacetylase
IQR: Interquartile Range
LEDD: Levodopa-Equivalent Daily Dose
MAOI: Monoamine Oxidase Inhibitor
MMSE: Mini-mental State Examination
PD: Parkinson’s disease
RBD: Rapid eye movement Sleep Behavior Disorder
RBDQ-HK: Rapid eye movement Sleep Behavior Disorder Questionnaire Hong Kong
SCFAs: Short-chain fatty acids
UPDRs: Unified Parkinson’s Disease Rating Scale
