# Supplementary material for: Serum short-chain fatty acids and its correlation with motor and non-motor symptoms in Parkinson’s disease patients
Source: BMC Neurol. 2022 Jan 7;22:13. doi: 10.1186/s12883-021-02544-7 (PMC8740341; doi:10.1186/s12883-021-02544-7)
Supplement: Supplementary file 1 — Additional file 1: Supp Table 1. The correlation between serum SCFAs and UPDRS part III score. Supplementary Fig. 1. UPDRS part III score was significantly positively correlated with disease duration in PD patients (n = 50), R = 0.494, P = 0.000. Investigated by Spearman nonparametric correlation analysis method. UPDRS, Unified Parkinson’s Disease Rating Scale. Supplementary Fig. 2. MMSE score was significantly negatively correlated with HAMD score in PD patients, R = -0.375, P = 0.007. Investigated by Spearman nonparametric correlation analysis method. MMSE, Mini-mental State Examination. HAMD, Hamilton Depression Scale. [file 12883_2021_2544_MOESM1_ESM.docx]

**Supplementary materials**

| Supp Table 1.The correlation between serum SCFAs and UPDRS part III score | | | |
| --- | --- | --- | --- |
|  | Correlation coefficient | *P* value | |
| Acetic acid | -0.232 | 0.105 | |
| Propionic acid | -0.365** | 0.009 | |
| Butyric acid | -0.246 | 0.084 | |
| Isobutyric acid | -0.095 | 0.512 | |
| Isovaleric acid | -0.089 | 0.539 | |
| Pentanoic acid | -0.015 | 0.917 | |
| Isocaproic acid | -0.200 | 0.163 | |
| Caproic acid | -0.183 | 0.203 | |
| Heptanoic acid | -0.145 | 0.314 | |
| *P* values was obtained from Spearman nonparametric correlation analysis method. **, *P*<0.01. | | |  |


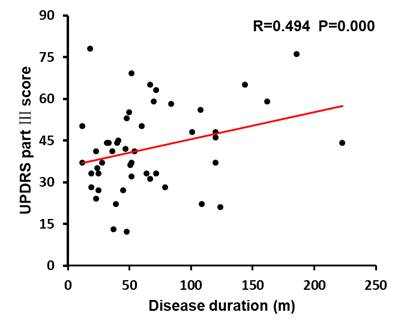


Supplementary Fig. 1. UPDRS part III score was significantly positively correlated with disease duration in PD patients (n=50), R=0.494, *P*=0.000. Investigated by Spearman nonparametric correlation analysis method. UPDRS, Unified Parkinson’s Disease Rating Scale.


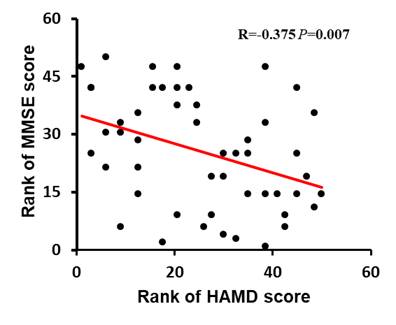


Supplementary Fig. 2. MMSE score was significantly negatively correlated with HAMD score in PD patients, R=-0.375, *P*=0.007. Investigated by Spearman nonparametric correlation analysis method. MMSE, Mini-mental State Examination. HAMD, Hamilton Depression Scale.
